# Drinking Patterns, Sociodemographic Correlates and Sex Differences Among Ghanaians Aged 15–49 Years: A Cross‐Sectional Study

**DOI:** 10.1002/puh2.70407

**Published:** 2026-10-05

**Authors:** Christian Chukwuma Ndu, Chioma Shallom Ndu, Christa Osei‐Mensah, Adusei Bofa, Georgina Afoakwah

**Affiliations:** ^1^ Department of Nursing and Midwifery Garden City University College Kumasi Ghana; ^2^ Department of Physician Assistantship Garden City University College Kumasi Ghana; ^3^ Department of Business Studies Garden City University College Kumasi Ghana

**Keywords:** alcohol, drinking patterns, Ghana, interaction, sex differences

## Abstract

**Background:**

Alcohol use varies by sex, but studies in Ghana have not formally tested whether sociodemographic correlates of drinking patterns differ between males and females. This study examined drinking patterns, their sociodemographic correlates and sex differences among Ghanaians aged 15–49 years.

**Methods:**

Dataset from 21,291 Ghanaians aged 15–49 years in the 2022 Ghana Demographic and Health Survey was analysed. Drinking patterns of respondents were classified on the basis of self‐reported quantity per drinking day. Multinomial logistic regression was used to estimate the adjusted odds ratios (AOR) accounting for sociodemographic confounders. Sex differences in sociodemographic correlates were tested using a single combined model with sex‐interaction terms.

**Results:**

Overall, prevalence of abstinence, moderate, low‐risk and heavy drinking were 81.6%, 11.3%, 4.1% and 3.0%, respectively. Males were strongly associated with moderate (AOR = 3.235; 95% CI = 2.801, 3.737), low‐risk (AOR = 1.809; 95% CI = 1.389, 2.355) and heavy drinking (AOR = 3.910; 95% CI = 2.881, 5.305). Older age, parenthood and employment increased odds of drinking patterns; marriage was protective. Significant sex interactions for heavy drinking versus abstinence contrast were found for age (*p* < 0.001), education (*p* < 0.001), marital status (*p* < 0.001), wealth (*p* = 0.009), parenthood (*p* < 0.001) and employment (*p* < 0.001).

**Conclusion:**

This study found that 18.4% of Ghanaians aged 15–49 years drink alcohol in varying patterns. Sociodemographic correlates of heavy drinking differ significantly between males and females. Sex‐stratified estimates suggest similar patterns for other drinking categories, though these were not formally tested. This supports the need for tailored, sex‐sensitive alcohol interventions.

## Graphical abstract

Prevalence of alcohol use among Ghanaians aged 15–49 years is 18.4%, with prevalence of all drinking patterns being higher in males than females. Sex differences were found in the sociodemographic correlates of drinking patterns, highlighting the need for sex‐sensitive interventions that minimise alcohol use among Ghanaians.

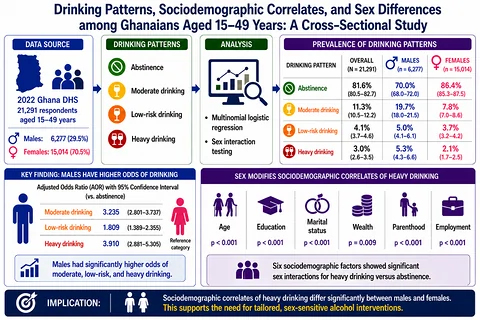

**Abbreviations**
DALYs: disability‐adjusted life years
GDHS: Ghana Demographic and Health Survey
SDGs: Sustainable Development Goals
WHO: World Health Organization

## 1 Introduction

Globally, in 2017, an estimated 47% of the global adult population consumed alcohol, resulting in a global adult per capita consumption of 6.5 L and 2.8 million deaths [1, 2]. By 2030, the global prevalence and adult per capita consumption are projected to increase to 50% and 7.6 L, respectively [1].

Regional differences in alcohol consumption have been identified, with the highest prevalence being for high sociodemographic index (SDI) countries and the lowest prevalence being for low‐to‐middle SDI countries [3]. In the European region, the prevalence of alcohol consumption has been reported to be 62.3% [4], with that of West Africa being reported to be 44% [5].

Alcohol use also varies by sex. Prevalence and patterns of alcohol use differ between males and females, with males having higher prevalence and engaging in higher risk drinking patterns as compared to females [2, 3, 5, 6, 7]. These higher patterns of alcohol use among males are associated with higher alcohol‐attributable deaths and disease burden in males [3].

In Ghana, prevalence of alcohol use among males and females has been estimated to be 48.7% and 17.5%, respectively [8, 9]. Alcohol per capita consumption is 7.4 L for males and 1.8 L for females, with alcohol‐attributable disability‐adjusted life years (DALYs) lost per 100,000 population being 3921 DALYs for males and 856 DALYs for females [2]. Alcohol use negatively affects the ability of countries to attain 13 out of the 17 Sustainable Development Goals (SDGs), more specifically SDG 3, which focuses on good health and well‐being [4].

Despite these descriptive sex differences, existing studies in Ghana have largely examined correlates of drinking separately for males and females or have only adjusted for sex as a covariate without formally assessing whether the effect of a sociodemographic factor differs significantly between sexes [8, 9, 10, 11, 12]. Consequently, these approaches cannot determine whether sociodemographic factors have different influences on drinking patterns for males and females. Moreover, most prior studies used binary drinking outcomes, overlooking important distinctions between drinking patterns and limiting the development of targeted, sex‐sensitive interventions. Understanding why these sex differences exist requires a theoretical lens that accounts for the social contexts in which males and females drink.

The Social Role Theory provides this lens, postulating that gender differences in behaviour arise from the varying social roles that men and women occupy in society [13]. When applied to alcohol use, this framework suggests that sociodemographic characteristics carry different social meanings and expectations for males and females and may potentially influence drinking behaviours that differ across sexes. This theoretical perspective, therefore, necessitates the formal testing of sex‐by‐sociodemographic interactions instead of the mere adjustment for sex as a confounder.

To address these gaps, this study conducted a secondary analysis of a large, nationally representative survey of Ghanaians aged 15–49 years and sought to:

1. Determine the prevalence of alcohol drinking patterns (abstinence, moderate, low‐risk and heavy drinking) among Ghanaians aged 15–49 years.
2. Identify sociodemographic correlates of drinking patterns among Ghanaians aged 15–49 years.
3. Determine sex differences in sociodemographic correlates of drinking patterns among Ghanaians aged 15–49 years.

The findings of this study will inform sex‐sensitive alcohol policy and intervention design in Ghana and contribute to the broader literature on sex and alcohol use in sub‐Saharan Africa.

## 2 Methods

### 2.1 Study Design

This study employed a quantitative cross‐sectional design using secondary data from the 2022 Ghana Demographic and Health Survey (GDHS). The dataset was derived from a nationwide household survey of male and female participants conducted by the Ghana Statistical Service between 17 October 2022 and 14 January 2023.

### 2.2 Study Area

This study was conducted in Ghana, a country situated in West Africa, that covers a total land area of 238,537 km^2^. According to 2021 estimates, Ghana has a total population of 30,832,019, of which males and females constituted 49.3% and 50.7%, respectively [14]. The GDHS sampling frame covers the entire country, including urban and rural areas.

### 2.3 Study Population

The target population was Ghanaian males and females aged 15–49 years who participated in the 2022 GDHS. According to the 2021 Population and Housing Census, the estimated population in this age range was approximately 15 million persons [14]. The survey population comprised 15,014 females aged 15–49 years and 7044 males aged 15–59 years who participated in the 2022 GDHS. For the purposes of this study, the final analytical sample was restricted to ages 15–49 years. Hence, the final analytical sample included 15,014 females and 6277 males aged 15–49 years, amounting to a total of 21,291 individuals.

### 2.4 Data Collection Instrument

GDHS structured questionnaires were administered by trained field staff through face‐to‐face interviews. The interviews were conducted in the participant's preferred local language. In order to ensure cross‐linguistic validity, the GDHS employed a standardised forward‐translation and back‐translation protocol across major Ghanaian languages, with field pre‐testing conducted to verify the conceptual equivalence prior to full deployment [15].

### 2.5 Inclusion Criteria

The study included all male and female respondents aged 15–49 years who were present in the 2022 GDHS dataset. Literacy was not a criterion for inclusion. The standard DHS protocol involved translation and back‐translation of the survey instrument across major Ghanaian languages [15].

### 2.6 Exclusion Criteria

The GDHS sampling frame excludes individuals below 15 years and above 49 years, as well as those who declined participation or were critically ill at the time of data collection. No additional exclusions were applied.

### 2.7 Sampling Technique and Sample Size

Publicly available GHIR8AFL and GHMR8AFL datasets from the 2022 GDHS were used in this study. The GDHS employs a stratified, two‐stage cluster sampling design to produce a nationally representative sample. The two‐stage cluster sampling involved selecting enumeration areas in the first stage and then households in the second. No separate calculation of sample size was required in this study because of the rigorous sampling framework employed by GDHS. A total of 21,291 respondents were interviewed: 6277 males (29.5%) and 15,014 females (70.5%). The higher proportion of females as compared to males is a reflection of the GDHS sampling design, where female questionnaire was administered to all eligible women in the selected households, whereas male questionnaire was administered to only one randomly selected man per household. The incorporation of GDHS sample weights in all analyses accounted for the differential probabilities of selection. After merging and checking for missing data on the selected variables, no missing values were found; thus, the analytical sample comprised all 21,291 respondents (100% retention).

### 2.8 Dependent Variables

The outcome variable was alcohol drinking patterns, classified on the basis of self‐reported usual quantity of alcohol consumed on an average drinking day in the last 30 days. The questions used to determine alcohol use were drawn from the standardised DHS module, which has been employed in nationally representative surveys in over 90 countries and has been used in peer‐reviewed studies examining alcohol use in Ghana [9]. Categories were adapted from the National Institute on Alcohol Abuse and Alcoholism (NIAAA) guidelines [16], though the GDHS did not standardise the definition of a ‘standard drink’. Drinking patterns were defined as

- Abstainers (coded 0): No alcohol consumption in the past 30 days.
- Moderate drinkers (coded 1): Males consuming 1–2 drinks and females consuming 1 drink on an average drinking day.
- Low‐risk drinkers (coded 2): Males consuming 3–4 drinks and females consuming 2–3 drinks on an average drinking day.
- Heavy drinkers (coded 3): Males consuming ≥5 drinks and females consuming ≥4 drinks on an average drinking day.

### 2.9 Independent Variables

The independent variables included as follows:

- Sex: Male, female
- Age: 15–19, 20–24, 25–29, 30–34, 35–39, 40–44 and 45–49 years
- Type of residence: Urban, rural
- Level of education: No education, primary, secondary and higher
- Wealth quintile: This variable is a composite score derived via a principal component analysis of household assets and living condition indicators (such as water source, sanitation facility, flooring material and ownership of consumer goods). Respondents were ranked and divided into five equal quintiles (poorest, poorer, middle, richer and richest)
- Marital status: Never in a union, married, living with partner, widowed, divorced and separated
- Currently working: No, yes
- Parent: Derived from the ‘total children ever born’ variable; male and female respondents reporting at least one child were classified as parents [15].

All independent variables were selected on the basis of a conceptual framework informed by prior literature and were included as confounders in all multivariable models.

### 2.10 Data Analysis

Participant characteristics and prevalence of drinking patterns were summarised using weighted frequencies and percentages, which are reported in tables. Bivariate associations between independent variables and drinking patterns were assessed using chi‐square tests. Multinomial logistic regression was used to estimate crude odds ratios (COR) and adjusted odds ratios (AOR) with 95% confidence intervals (CI) for each drinking pattern category versus abstainers. All confounders were included a priori in the multivariable models. The study also conducted sex‐stratified analyses in order to assess sex‐specific odds ratios.

To test for sex differences in sociodemographic correlates, this study used a single combined multinomial logistic regression model that included all main effects plus interaction terms between sex and each sociodemographic variable. Interaction *p* values were obtained from the Wald tests for the interaction terms corresponding to the heavy drinking versus abstainer contrast, as this was the primary pattern of interest. This approach directly tests whether the effect of each sociodemographic factor on heavy drinking differs significantly between males and females, while adjusting for all other confounders. For all other drinking patterns, interaction was presented descriptively via sex‐stratified estimates, without formal computation of interaction *p* values.

The multinomial regression model converged successfully; the likelihood ratio test comparing the final model to a null model was significant (*p* < 0.001), indicating good overall fit. Multicollinearity was evaluated using variance inflation factors (all VIF < 2.5). All statistical analyses incorporated GDHS sample weights (v005 for females; mv005 for males, divided by 1,000,000 as per GDHS convention), primary sampling units (v021/mv021) and stratification (v022/mv022) using the Complex Samples module in SPSS version 27. Statistical significance was set at *p* < 0.05.

### 2.11 Ethical Consideration

This study involved secondary analysis of a de‐identified, publicly available 2022 GDHS dataset; no personal identifiers were accessed. The original GDHS protocol received ethical approval from the Ethical Review Committee of the Ghana Health Service and the ICF Institutional Review Board. All GDHS procedures complied with the ethical standards of these committees as well as the US and international ethical research standards, and the Declaration of Helsinki. Written informed consent was obtained from all participants by the GDHS programme prior to data collection [15]. Because this study analysed fully anonymised secondary data, no additional ethical approval was required as per institutional policy. Data access was granted by the Ghana Statistical Service under standard data use terms.

## 3 Results

Analysis of data from 21,291 Ghanaians aged 15–49 years revealed a population in which alcohol abstinence is the dominant pattern, yet considerable variation exists in the type and intensity of drinking among those who consume alcohol. The following sections present the sociodemographic profile of participants, the prevalence and distribution of drinking patterns, their adjusted sociodemographic correlates and the extent to which those correlates differ formally between males and females.

### 3.1 Characteristics of Study Participants

As shown in Table 1, the majority of participants were female (70.5%), employed (77.0%), parents (62.0%), had secondary education (61.0%) and resided in urban areas (56.4%). Regarding age and marital status, the largest subgroups were those aged 15–19 years (19.3%) and those never in a union (39.8%).

**TABLE 1:** Characteristics of study participants (*N* = 21,291).

Characteristics	Weighted frequency (*N*)	Weighted percentage
Sex		
Female	15,014	70.5
Male	6277	29.5
Age		
15–19	4107	19.3
20–24	3728	17.5
25–29	3227	15.2
30–34	3105	14.6
35–39	2867	13.5
40–44	2388	11.2
45–49	1869	8.8
Residence		
Urban	12,000	56.4
Rural	9291	43.6
Educational level		
No education	3038	14.3
Primary	2795	13.1
Secondary	12,990	61.0
Higher	2468	11.6
Marital status		
Never in a union	8476	39.8
Married	8381	39.4
Living with partner	2651	12.5
Widowed	385	1.8
Divorced	472	2.2
Separated	926	4.4
Wealth		
Poorest	3535	16.6
Poorer	3845	18.1
Middle	4259	20.0
Richer	4844	22.8
Richest	4808	22.6
Parent		
No	8086	38.0
Yes	13,205	62.0
Currently working		
No	4895	23.0
Yes	16,396	77.0

### 3.2 Prevalence of Drinking Patterns

Overall prevalence of abstinence, moderate, low‐risk and heavy drinking was 81.6%, 11.3%, 4.1% and 3.0%, respectively (Table 2). Abstinence decreased with increasing age, whereas low‐risk and heavy drinking increased with age. Widowed (9.0%) and separated (7.0%) participants had the highest prevalence of low‐risk drinking and heavy drinking, respectively. Parents and employed participants had higher prevalence of all drinking patterns than their counterparts. Bivariate associations (chi‐square) were significant for all variables except type of residence (*p* = 0.497), wealth (*p* = 0.283) and educational level (*p* = 0.211).

**TABLE 2:** Prevalence of drinking patterns among Ghanaians aged 15–49 years (*N* = 21,291).

Characteristics	Abstainers		Moderate		Low risk		Heavy		*p* value
	*n*	% (CI)	*n*	% (CI)	*n*	% (CI)	*n*	% (CI)	
Overall	17,373	81.6 (80.5, 82.7)	2406	11.3 (10.5, 12.2)	870	4.1 (3.7, 4.6)	642	3.0 (2.6, 3.5)	
Sex									<0.001
Female	12,977	86.4 (85.3, 87.5)	1171	7.8 (7.0, 8.6)	557	3.7 (3.2, 4.2)	309	2.1 (1.7, 2.5)	
Male	4396	70.0 (68.0, 72.0)	1235	19.7 (18.0, 21.5)	313	5.0 (4.1, 6.1)	333	5.3 (4.3, 6.6)	
Age									<0.001
15–19	3686	89.8 (88.2, 91.2)	321	7.8 (6.6, 9.2)	68	1.7 (1.2, 2.3)	30	0.7 (0.5, 1.1)	
20–24	3135	84.1 (82.1, 85.9)	411	11.0 (9.5, 12.7)	135	3.6 (2.8, 4.7)	48	1.3 (0.9, 1.9)	
25–29	2655	82.2 (80.2, 84.1)	337	10.4 (9.1, 12.0)	144	4.5 (3.6, 5.5)	92	2.9 (2.2, 3.7)	
30–34	2447	78.8 (76.5, 80.9)	404	13.0 (11.4, 14.9)	147	4.7 (3.8, 5.9)	107	3.4 (2.5, 4.7)	
35–39	2234	77.9 (75.7, 80.0)	363	12.7 (11.1, 14.4)	150	5.2 (4.2, 6.4)	120	4.2 (3.2, 5.5)	
40–44	1801	75.4 (73.1, 77.5)	340	14.2 (12.3, 16.3)	126	5.3 (4.3, 6.5)	122	5.1 (3.9, 6.6)	
45–49	1415	75.7 (72.8, 78.4)	230	12.3 (10.4, 14.5)	100	5.4 (4.2, 6.8)	123	6.6 (5.0, 8.7)	
Residence									0.497
Urban	9838	82.0 (80.6, 83.3)	1297	10.8 (9.8, 11.9)	486	4.0 (3.5, 4.7)	379	3.2 (2.5, 4.0)	
Rural	7535	81.1 (79.2, 82.8)	1109	11.9 (10.6, 13.4)	384	4.1 (3.6, 4.8)	263	2.8 (2.4, 3.4)	
Educational level									0.211
No education	2527	83.2 (80.3, 85.7)	302	9.9 (8.2, 12.0)	120	4.0 (3.2, 4.9)	89	2.9 (2.2, 4.0)	
Primary	2222	79.5 (77.3, 81.5)	346	12.4 (10.7, 14.2)	128	4.6 (3.6, 5.8)	99	3.5 (2.7, 4.6)	
Secondary	10,621	81.8 (80.5, 82.9)	1444	11.1 (10.2, 12.1)	526	4.0 (3.6, 4.6)	399	3.1 (2.5, 3.7)	
Higher	2003	81.2 (78.6, 83.5)	314	12.7 (10.8, 14.9)	96	3.9 (2.9, 5.2)	55	2.2 (1.5, 3.5)	
Marital status									<0.001
Never in a union	7114	83.9 (82.4, 85.3)	913	10.8 (9.7, 11.9)	294	3.5 (2.8, 4.2)	157	1.9 (1.5, 2.3)	
Married	6880	82.1 (80.5, 83.6)	883	10.5 (9.5, 11.7)	323	3.9 (3.3, 4.5)	294	3.5 (2.8, 4.4)	
Living with partner	2030	76.6 (74.0, 79.0)	404	15.2 (13.2, 17.6)	126	4.8 (3.8, 6.0)	90	3.4 (2.4, 4.7)	
Widowed	311	80.8 (75.0, 85.5)	26	6.8 (4.3, 10.5)	35	9.0 (5.8, 13.8)	13	3.3 (1.4, 7.8)	
Divorced	347	73.5 (67.5, 78.7)	63	13.4 (9.7, 18.2)	38	8.1 (5.4, 11.9)	24	5.1 (2.8, 8.9)	
Separated	691	74.6 (70.4, 78.4)	117	12.7 (10.0, 16.0)	54	5.7 (4.2, 7.8)	64	7.0 (5.1, 9.6)	
Wealth									0.283
Poorest	2855	80.8 (77.4, 83.7)	410	11.6 (9.5, 14.0)	165	4.7 (3.8, 5.7)	106	3.0 (2.3, 3.9)	
Poorer	3214	83.6 (81.5, 85.5)	395	10.3 (8.9, 11.8)	125	3.2 (2.6, 4.1)	111	2.9 (2.3, 3.7)	
Middle	3503	82.2 (80.4, 84.0)	463	10.9 (9.6, 12.4)	176	4.1 (3.3, 5.1)	116	2.7 (2.2, 3.5)	
Richer	3887	80.2 (77.9, 82.4)	612	12.6 (11.1, 14.4)	177	3.7 (2.9, 4.6)	169	3.5 (2.5, 4.9)	
Richest	3914	81.4 (79.5, 83.2)	526	11.0 (9.4, 12.7)	227	4.7 (3.9, 5.7)	140	2.9 (2.0, 4.2)	
Parent									<0.001
No	6842	84.6 (83.1, 86.0)	868	10.7 (9.7, 11.9)	262	3.2 (2.6, 4.0)	113	1.4 (1.1, 1.8)	
Yes	10,531	79.7 (78.4, 81.0)	1538	11.6 (10.7, 12.7)	608	4.6 (4.1, 5.2)	529	4.0 (3.4, 4.7)	
Currently working									<0.001
No	4424	90.4 (89.1, 91.5)	301	6.2 (5.3, 7.1)	111	2.3 (1.7, 2.9)	60	1.2 (0.8, 1.8)	
Yes	12,949	79.0 (77.7, 80.2)	2105	12.8 (11.9, 13.9)	759	4.6 (4.1, 5.2)	582	3.6 (3.0, 4.2)	

*Note: n*—weighted frequency of variable; %—weighted percentage of frequency.

Abbreviation: CI, confidence interval.

### 3.3 Sociodemographic Correlates of Drinking Patterns

After adjusting for all confounders, compared to females, males had significantly higher odds of engaging in all drinking patterns (Table 3). As compared to participants aged 15–19 years, all age groups had significantly higher odds of engaging in all drinking patterns, with odds mostly increasing with age. Participants aged 40–44 years had the highest odds of moderate drinking (AOR = 2.354; 95% CI = 1.695, 3.269), whereas those aged 45–49 years had the highest odds of low‐risk (AOR = 4.743; 95% CI = 2.805, 8.019) and heavy drinking (AOR = 10.777; 95% CI = 5.420, 21.428). Participants with primary education had significantly higher odds of moderate drinking (AOR = 1.262; 95% CI = 1.008, 1.579). Employed participants had significantly higher odds of moderate (AOR = 1.750; 95% CI = 1.467, 2.087) and low‐risk drinking (AOR = 1.475; 95% CI = 1.094, 1.990). Parenthood was significantly associated with higher odds of heavy drinking (AOR = 2.309; 95% CI = 1.450, 3.678). Married participants had significantly lower odds of all drinking patterns, and poorer participants had significantly lower odds of low‐risk drinking (AOR = 0.614; 95% CI = 0.439, 0.857).

**TABLE 3:** Sex‐adjusted multinomial logistic regression of sociodemographic correlates of drinking patterns (*N* = 21,291).

Characteristics	Crude				Adjusted			
	*p* value	Moderate (vs. abstinence)	Low risk (vs. abstinence)	Heavy (vs. abstinence)	*p* value	Moderate (vs. abstinence)	Low risk (vs. abstinence)	Heavy (vs. abstinence)
		COR (95% CI)	COR (95% CI)	COR (95% CI)		AOR (95% CI)	AOR (95% CI)	AOR (95% CI)
Sex	<0.001				<0.001			
Female		1	1	1		1	1	1
Male		3.112 (2.728, 3.550)	1.662 (1.284, 2.152)	3.175 (2.368, 4.256)		3.235 (2.801, 3.737)	1.809 (1.389, 2.355)	3.910 (2.881, 5.305)
Age	<0.001				<0.001			
15–19		1	1	1		1	1	1
20–24		1.503 (1.212, 1.864)	2.314 (1.585, 3.377)	1.869 (1.102, 3.170)		1.447 (1.128, 1.857)	2.454 (1.628, 3.697)	1.885 (1.096, 3.243)
25–29		1.456 (1.169, 1.813)	2.918 (2.047, 4.159)	4.262 (2.568, 7.075)		1.397 (1.054, 1.852)	3.346 (2.150, 5.209)	4.249 (2.339, 7.716)
30–34		1.896 (1.524, 2.360)	3.233 (2.197, 4.758)	5.359 (3.091, 9.290)		1.944 (1.447, 2.612)	4.129 (2.454, 6.948)	5.501 (2.595, 11.662)
35–39		1.865 (1.495, 2.328)	3.604 (2.459, 5.283)	6.599 (3.905, 11.150)		2.019 (1.473, 2.769)	4.722 (2.899, 7.690)	6.768 (3.514, 13.036)
40–44		2.163 (1.731, 2.703)	3.752 (2.554, 5.512)	8.324 (5.254, 13.188)		2.354 (1.695, 3.269)	4.704 (2.758, 8.023)	8.074 (4.021, 16.213)
45–49		1.861 (1.448, 2.390)	3.811 (2.595, 5.595)	10.728 (6.395, 17.999)		2.067 (1.450, 2.947)	4.743 (2.805, 8.019)	10.777 (5.420, 21.428)
Residence	0.555				0.284			
Urban		1	1	1		1	1	1
Rural		1.117 (0.936, 1.333)	1.034 (0.821, 1.301)	0.907 (0.663, 1.241)		1.178 (0.960, 1.445)	1.093 (0.840, 1.423)	0.864 (0.621, 1.202)
Educational level	0.097							
No education		1	1	1	<0.001	1	1	1
Primary		1.304 (1.036, 1.642)	1.215 (0.872, 1.693)	1.260 (0.838, 1.894)		1.262 (1.008, 1.579)	1.347 (0.982, 1.847)	1.464 (0.954, 2.248)
Secondary		1.139 (0.908, 1.428)	1.041 (0.805, 1.346)	1.062 (0.755, 1.494)		1.098 (0.891, 1.353)	1.231 (0.933, 1.624)	1.322 (0.916, 1.908)
Higher		1.312 (0.988, 1.743)	1.007 (0.696, 1.457)	0.783 (0.457, 1.344)		1.093 (0.828, 1.443)	0.856 (0.572, 1.283)	0.724 (0.433, 1.210)
Marital status	<0.001				<0.001			
Never in a union		1	1	1		1	1	1
Married		1.002 (0.868, 1.155)	1.137 (0.874, 1.478)	1.938 (1.476, 2.545)		0.589 (0.471, 0.738)	0.493 (0.343, 0.709)	0.407 (0.263, 0.630)
Living with partner		1.552 (1.279, 1.883)	1.511 (1.105, 2.065)	2.000 (1.347, 2.971)		1.264 (0.991, 1.612)	0.863 (0.586, 1.270)	0.706 (0.426, 1.170)
Widowed		0.655 (0.402, 1.068)	2.705 (1.618, 4.522)	1.876 (0.753, 4.673)		0.528 (0.312, 0.895)	1.299 (0.710, 2.375)	0.466 (0.174, 1.247)
Divorced		1.420 (0.973, 2.073)	2.655 (1.662, 4.241)	3.126 (1.671, 5.845)		0.924 (0.589, 1.448)	1.126 (0.684, 1.852)	0.644 (0.321, 1.292)
Separated		1.324 (0.989, 1.771)	1.857 (1.244, 2.771)	4.253 (2.910, 6.218)		1.014 (0.723, 1.424)	0.963 (0.598, 1.551)	1.236 (0.746, 2.047)
Wealth	0.213				0.099			
Poorest		1	1	1		1	1	1
Poorer		0.856 (0.664, 1.103)	0.670 (0.483, 0.931)	0.937 (0.643, 1.364)		0.833 (0.643, 1.079)	0.614 (0.439, 0.857)	0.818 (0.556, 1.204)
Middle		0.922 (0.704, 1.206)	0.869 (0.625, 1.210)	0.899 (0.619, 1.306)		0.950 (0.717, 1.259)	0.791 (0.555, 1.129)	0.756 (0.504, 1.136)
Richer		1.097 (0.836, 1.439)	0.788 (0.561, 1.108)	1.174 (0.735, 1.874)		1.108 (0.813, 1.510)	0.705 (0.486, 1.023)	0.909 (0.561, 1.473)
Richest		0.939 (0.709, 1.242)	0.998 (0.733, 1.358)	0.969 (0.604, 1.553)		1.018 (0.742, 1.397)	0.998 (0.673, 1.479)	0.864 (0.516, 1.446)
Parent	<0.001				0.006			
No		1	1	1		1	1	1
Yes		1.151 (1.018, 1.300)	1.506 (1.181, 1.920)	3.038 (2.331, 3.959)		1.154 (0.918, 1.451)	1.139 (0.787, 1.648)	2.309 (1.450, 3.678)
Currently working	<0.001				<0.001			
No		1	1	1		1	1	1
Yes		2.387 (2.036, 2.797)	2.347 (1.770, 3.111)	3.341 (2.239, 4.986)		1.750 (1.467, 2.087)	1.475 (1.094, 1.990)	1.284 (0.832, 1.983)

Abbreviations: AOR, adjusted odds ratio; CI, confidence interval; COR, crude odds ratio.

### 3.4 Sex Differences in Sociodemographic Correlates of Drinking Patterns

A single combined model with sex and covariate terms was used in testing interactions for the heavy drinking versus abstinence contrast. All sociodemographic correlates, with the exception of type of residence (*p* = 0.593), showed significant sex differences (Table 4). The sex differences in moderate and low‐risk drinking were described on the basis of sex‐stratified estimates rather than formal interaction tests. In terms of age, males of all age groups had significantly higher odds of engaging in all drinking patterns, with odds of heavy drinking being considerably higher than those of other drinking patterns. Females of all age groups, however, only had significantly higher odds of low‐risk drinking, with females aged 35–49 years being the only age group to have significantly higher odds of heavy drinking. Among males, participants with primary and secondary education had significantly higher odds of moderate and heavy drinking, but no significant association was found between education and drinking pattern among females. Marriage was protective against heavy drinking in both sexes, but the effect appeared more pronounced in females (AOR = 0.311; 95% CI = 0.174, 0.558) than in males (AOR = 0.459; 95% CI = 0.236, 0.891). Wealth showed no association with heavy drinking in men, but poorer (AOR = 0.558; 95% CI = 0.386, 0.806), middle (AOR = 0.619; 95% CI = 0.426, 0.900) and richer (AOR = 0.498; 95% CI = 0.327, 0.758) females were associated with lower odds of low‐risk drinking. Parenthood increased odds of heavy drinking in both males (AOR = 2.494; 95% CI = 1.304, 4.771) and females (AOR = 2.162; 95% CI = 1.052, 4.442), but odds of moderate drinking were only elevated among males (AOR = 1.811; 95% CI = 1.342, 2.445). Employment increased moderate drinking in both males (AOR = 1.746; 95% CI = 1.224, 2.489) and females (AOR = 1.767; 95% CI = 1.423, 2.195), but low‐risk drinking only in males (AOR = 2.333; 95% CI = 1.127, 4.830).

**TABLE 4:** Sex‐stratified multinomial regression analysis of sociodemographic correlates (*N* = 21,291).

Characteristics	Males				Females				Interaction *p* value
	*p* value	Moderate (vs. abstinence)	Low risk (vs. abstinence)	Heavy (vs. abstinence)	*p* value	Moderate (vs. abstinence)	Low risk (vs. abstinence)	Heavy (vs. abstinence)	
		AOR (95% CI)	AOR (95% CI)	AOR (95% CI)		AOR (95% CI)	AOR (95% CI)	AOR (95% CI)	
Age	<0.001				<0.001				<0.001
15–19		1	1	1		1	1	1	
20–24		2.048 (1.436, 2.920)	3.277 (1.857, 5.783)	3.548 (1.200, 10.492)		1.117 (0.794, 1.571)	2.070 (1.212, 3.536)	1.219 (0.565, 2.630)	
25–29		1.963 (1.296, 2.974)	4.672 (2.485, 8.784)	10.316 (3.380, 31.482)		1.094 (0.737, 1.623)	2.817 (1.594, 4.981)	2.357 (0.955, 5.817)	
30–34		2.783 (1.791, 4.323)	5.803 (2.684, 12.545)	13.380 (4.098, 43.688)		1.476 (0.977, 2.230)	3.452 (1.723, 6.914)	3.057 (0.941, 9.937)	
35–39		3.298 (2.087, 5.209)	8.940 (3.913, 20.426)	20.075 (6.610, 60.968)		1.330 (0.846, 2.092)	3.333 (1.828, 6.079)	3.100 (1.095, 8.781)	
40–44		3.032 (1.942, 4.735)	8.674 (3.710, 20.280)	11.838 (3.886, 36.064)		1.819 (1.150, 2.875)	3.197 (1.600, 6.387)	6.593 (2.257, 19.256)	
45–49		3.047 (1.828, 5.080)	7.205 (2.911, 17.833)	32.795 (9.927, 108.338)		1.488 (0.930, 2.379)	3.795 (1.923, 7.489)	4.194 (1.533, 11.473)	
Residence	0.253				0.764				0.593
Urban		1	1	1		1	1	1	
Rural		1.217 (0.949, 1.561)	1.040 (0.655, 1.652)	0.717 (0.423, 1.214)		1.127 (0.864, 1.471)	1.134 (0.828, 1.555)	1.017 (0.669, 1.546)	
Educational level	0.007				0.009				<0.001
No education		1	1	1		1	1	1	
Primary		1.657 (1.215, 2.259)	1.937 (1.070, 3.508)	2.332 (1.269, 4.284)		1.067 (0.791, 1.439)	1.150 (0.800, 1.654)	1.067 (0.636, 1.792)	
Secondary		1.528 (1.147, 2.034)	1.183 (0.700, 2.000)	1.757 (1.066, 2.894)		0.796 (0.609, 1.042)	1.209 (0.870, 1.680)	1.038 (0.630, 1.710)	
Higher		1.276 (0.894, 1.820)	0.883 (0.464, 1.681)	1.027 (0.546, 1.933)		0.925 (0.620, 1.380)	0.768 (0.474, 1.242)	0.426 (0.175, 1.040)	
Marital status	<0.001				<0.001				<0.001
Never in a union		1	1	1		1	1	1	
Married		0.531 (0.385, 0.731)	0.349 (0.181, 0.672)	0.459 (0.236, 0.891)		0.524 (0.390, 0.705)	0.528 (0.338, 0.826)	0.311 (0.174, 0.558)	
Living with partner		1.437 (1.011, 2.043)	0.696 (0.339, 1.427)	0.846 (0.415, 1.724)		1.163 (0.860, 1.573)	0.982 (0.616, 1.564)	0.603 (0.303, 1.202)	
Widowed		0.685 (0.174, 2.701)	0.186 (0.020, 1.682)	0.125 (0.014, 1.133)		0.496 (0.271, 0.908)	1.715 (0.898, 3.277)	0.458 (0.164, 1.276)	
Divorced		0.494 (0.217, 1.125)	0.386 (0.148, 1.006)	0.786 (0.269, 2.293)		1.121 (0.651, 1.928)	1.608 (0.891, 2.903)	0.421 (0.169, 1.048)	
Separated		1.181 (0.680, 2.054)	0.498 (0.186, 1.335)	2.192 (0.968, 4.964)		0.946 (0.610, 1.466)	1.263 (0.736, 2.169)	0.856 (0.455, 1.611)	
Wealth	0.306				0.020				0.009
Poorest		1	1	1		1	1	1	
Poorer		0.951 (0.684, 1.323)	0.748 (0.421, 1.328)	0.794 (0.440, 1.433)		0.736 (0.535, 1.011)	0.558 (0.386, 0.806)	0.876 (0.542, 1.415)	
Middle		1.074 (0.753, 1.530)	1.299 (0.719, 2.346)	0.580 (0.309, 1.089)		0.844 (0.600, 1.189)	0.619 (0.426, 0.900)	1.016 (0.635, 1.626)	
Richer		1.198 (0.815, 1.761)	1.302 (0.751, 2.257)	0.841 (0.414, 1.706)		1.019 (0.699, 1.485)	0.498 (0.327, 0.758)	1.016 (0.586, 1.760)	
Richest		1.002 (0.676, 1.487)	1.235 (0.665, 2.294)	0.597 (0.287, 1.240)		1.002 (0.673, 1.491)	0.896 (0.554, 1.447)	1.363 (0.724, 2.564)	
Parent	<0.001				0.090				<0.001
No		1	1	1		1	1	1	
Yes		1.811 (1.342, 2.445)	1.876 (0.972, 3.620)	2.494 (1.304, 4.771)		0.831 (0.613, 1.126)	0.867 (0.568, 1.324)	2.162 (1.052, 4.442)	
Currently working	0.003				<0.001				<0.001
No		1	1	1		1	1	1	
Yes		1.746 (1.224, 2.489)	2.333 (1.127, 4.830)	1.024 (0.476, 2.200)		1.767 (1.423, 2.195)	1.354 (0.975, 1.879)	1.466 (0.854, 2.518)	

Abbreviations: AOR, adjusted odds ratio; CI, confidence intervals; COR, crude odds ratio.

In summary, these results paint a consistent picture where alcohol use in Ghana is predominantly male‐driven, shaped by life‐course factors and socially modulated by marriage and parenthood in ways that diverge markedly between sexes. Formal interaction testing confirmed that the associations of most sociodemographic characteristics with heavy drinking differ significantly between males and females. The patterns observed for moderate and low‐risk drinking from sex‐stratified estimates follow a broadly similar direction, though these were not subjected to formal interaction testing. The meaning of these findings in relation to existing evidence and their implications for intervention are examined in Section 4.

## 4 Discussion

This study provides novel evidence on the prevalence of drinking patterns and sociodemographic correlates of those patterns among Ghanaians aged 15–49 years, using nationally representative sample. The sociodemographic correlates of alcohol use in Ghana differed significantly between sexes, with males having a disproportionately higher burden relative to females. These findings are discussed below in the context of existing evidence.

### 4.1 Prevalence of Drinking Patterns

Using a large, nationally representative sample, we found that 81.6% of respondents abstained from alcohol, whereas 18.4% consumed alcohol in varying patterns. This overall prevalence is lower than the global (47.0%) and West African (44.0%) estimates [1, 5], reflecting possible under‐reporting due to social desirability or genuine cultural and religious norms in Ghana.

Among alcohol consumers, moderate drinking (61.4%) was the most prevalent pattern, followed by low‐risk (22.2%) and heavy drinking (16.4%). These proportions differ from those reported in a US study where 87.5% of current drinkers engaged in lighter drinking and only 12.5% in heavier drinking patterns [17]. Our finding suggests that when Ghanaians drink, they consume larger amounts per occasion. This is consistent with World Health Organization (WHO) data showing that drinkers in Africa consume the highest average daily amount (33.5 g/day) compared to the Americas (27.1 g/day) and Europeans (31.9 g/day), exceeding the global average of 26.7 g/day [2]. The WHO also reported that drinkers in lower‐middle‐income countries, to which Ghana belongs, consume more (30.9 g/day) than those in low‐ (25.1 g/day), upper‐middle‐ (23.6 g/day) and high‐income countries (28.3 g/day) [2].

### 4.2 Sociodemographic Correlates of Drinking Patterns

We found that sex, age, marital status, parenthood and employment were significantly associated with drinking patterns, after adjusting for all confounders. Compared to females, males were found to be significantly associated with higher odds of all patterns of alcohol use. This finding is consistent with previous studies which found that alcohol use by females is constrained by gender norms, highlighting sex and gender differences in alcohol use [1, 3, 18].

Older participants (35–49 years) had stronger associations with all drinking patterns compared to younger participants (20–34 years). This age‐specific variation may reflect different drivers: among younger adults, social interactions, anxiety, depression and impulsive decision‐making; and among older adults, life stress, marital status and childhood adversities [19].

Marriage was a protective factor against all patterns, with the protective effect strengthening as alcohol consumption severity increased. This is consistent with studies from the USA [20, 21] and may be explained by spouses being able to control the recreational outlets of their alcohol‐consuming partners [22].

Contrary to some previous reports that parents limit alcohol use to serve as role models [23, 24, 25], we found that parenthood was associated with higher odds of heavy drinking. This may reflect parenting stress or social context where alcohol is used as a coping mechanism, particularly among fathers.

Employment was associated with higher odds of moderate and low‐risk drinking, but not heavy drinking. The absence of an association between employment and heavy drinking possibly reflects differences in social context of alcohol use among employed individuals, although this study did not collect data on workplace policies, or organisational culture [26]. The relationship between occupational environment and drinking patterns warrants direct investigation in future studies.

Regarding education, only primary education was found to be associated with moderate drinking. There were no significant associations found for secondary or higher education. This non‐linear pattern in the association between education and drinking patterns is possibly explained by social network composition that differs across education levels in ways that shape drinking norms, but this explanation remains speculative as it requires data on social networks and alcohol‐related attitudes to examine properly.

### 4.3 Sex Differences in Sociodemographic Correlates

This study found important differences in the sociodemographic correlates of alcohol use across males and females, with statistically significant interaction effects between heavy drinking and abstinence observed for age, educational level, marital status, wealth, parenthood and employment status.

The most prominent sex difference was observed in age. The odds of heavy drinking were found to increase dramatically with advancing age among males, peaking in males aged 45–49 years, who had 32.8 times the odds of males aged 15–19 years. A less dramatic age‐dependent elevation of odds of heavy drinking was observed among females, with females aged 45–49 years having 4.2 times the odds of heavy drinking compared to their youngest counterparts. The significant interaction between age and sex suggests that males accumulate alcohol‐related risks across the life course, whereas females’ drinking remains more constrained by social norms [18, 27, 28].

Educational attainment demonstrated a significant association with alcohol consumption among males but not females. Unlike females, males with primary or secondary education had significantly higher odds of moderate and heavy drinking compared to those with no education. The positive association between education and drinking patterns may reflect pathways through which formal schooling increases social exposure, peer influence and access to alcohol [18, 29]. The negative education gradient observed in males may be indicative of differences in exposure to health information, social norms or economic circumstances, found within the participants of different education levels. The lack of association between education and drinking patterns found in females may reflect stronger social norm constraints on female drinking that override social pathways linked with education. These findings should, however, be interpreted cautiously, as they need to be confirmed by qualitative and longitudinal studies.

Marital status was significantly associated with drinking patterns for both sexes, and the interaction was also significant. Being married was protective against all drinking patterns for both males and females compared to those never in a union. However, among men, cohabitation was associated with elevated odds of moderate drinking, but no significant association was found among females. The protective effect of formal marriage may reflect social monitoring and health‐promoting behaviours associated with marital roles [20, 21]. This protection is, however, not replicated in males in informal unions.

Wealth was associated with significantly lower odds of alcohol use among females but not males, with a significant interaction. Among females, higher wealth quintiles were associated with lower odds of low‐risk drinking, with lower odds generally observed across higher wealth categories. This finding among females, which is consistent with a previous study on women in Ghana, suggests that economic resources may influence drinking behaviour by females through exposure to different social environments [9]. Wealthier females could also face stronger social sanctions against visible alcohol consumption [18]. The absence of wealth effect among males may reflect the broader social permissibility of male drinking across all economic strata, making wealth a less discriminating factor.

Parenthood was significantly associated with drinking patterns among males (*p* < 0.001), but the association was largely non‐significant among females (*p* = 0.090), yielding a significant interaction (*p* < 0.001). Among males, being a parent was associated with about 1.8 times the odds of moderate and 2.5 times the odds of heavy drinking compared to non‐parents, whereas among females, only heavy drinking showed association. These findings may reflect gendered stress responses to parenting demands, whereby males are more likely to use alcohol as a coping mechanism or to engage in social drinking outside the home, whereas females’ alcohol use is more heavily constrained by their primary caregiving roles [23, 24, 30, 31].

Finally, employment increased moderate drinking in both sexes, but low‐risk drinking increased only in males. This suggests the worker role influences drinking differently for males and females, likely reflecting gendered expectations [18]. The association between employment and moderate drinking, observed in both males and females, is potentially due to increased social opportunities and financial resources that come with being employed, and these ultimately enhance alcohol accessibility. For females, the lack of association with low‐risk and heavy drinking found merit further exploration, including examining the roles of workplace norms, gender‐specific social oversight or variations in social settings after work. These explanations, however, remain speculative until proven by studies that directly measure the influence of workplace policies or organisation culture on alcohol use in the Ghanaian context.

The sex‐interaction patterns identified in this study suggest that sociodemographic pathways influencing alcohol consumption vary between males and females. In line with the Social Role Theory, factors like employment, parenthood and marital status, which are associated with adult social roles, may have different influences of drinking behaviours based on sex. For males, employment increased low‐risk drinking, whereas parenthood increased moderate drinking. Additionally, marriage provided a strong protection against heavy drinking in females. These divergences suggest that alcohol consumption is possibly normalised or facilitated by male‐dominated social roles (e.g., breadwinner, social drinker), whereas female social roles (e.g., pregnancy, spouse) imposed stronger constraints [18, 27]. The significant education‐by‐sex interaction possibly indicates that the pathway through which education affects drinking is potentially shaped by gender‐specific social norms. These findings highlight the need for interventions that are explicitly tailored to the social contexts of males and females in Ghana, rather than assuming uniform effects of sociodemographic factors across sexes.

### 4.4 Strengths and Limitations

The generalisability of our findings is enhanced by the large, nationally representative sample; the use of complex survey design; the multinomial outcome distinguishing four drinking patterns; and the formal interaction tests for sex differences.

Limitations should be acknowledged. First, the cross‐sectional design limits causal inference. Second, alcohol use was self‐reported, which may be subject to recall bias and under‐reporting. Third, the GDHS did not standardise a ‘standard drink’; hence, a single ‘drink’ may differ in volume and alcohol content depending on the social, cultural and regional context within Ghana. This introduces measurement imprecision in the classification of drinking patterns, thereby limiting the comparability of the findings of the study with studies that employed the standardised definition of drink. Fourth, the survey excluded adults aged 50 years and older, limiting generalisability to older Ghanaians. Fifth, we could not adjust for unmeasured confounders, such as peer influence, cultural norms or alcohol accessibility.

### 4.5 Implications for Policy and Practice

Even light drinking is associated with adverse health effects, and heavier patterns increase risk substantially [3, 32, 33, 34]. Our findings have several implications. First, sex‐sensitive interventions are needed. Heavy drinking in older males aged 40–49 years should be a priority, and females’ alcohol use should be monitored, particularly among mothers who were found to show elevated odds of heavy drinking. Second, workplace alcohol policies should possibly focus on males, as they are more likely to engage in low‐risk drinking, which is associated with employment. The protective effect of marriage against all drinking patterns for both males and females suggests that couple‐based or family‐centred interventions could be effective. The insignificant effect of wealth on heavy drinking in males indicates that economic status alone does not drive risk drinking; however, normative and cultural factors could play more important roles in elevating risks.

## 5 Conclusion

This study found that 18.4% of Ghanaians aged 15–49 years consume alcohol, and drinkers tend to consume relatively large amounts. It should be noted, however, that the lack of standardised definition of ‘drink’ may affect the precision of pattern estimates. Formal interaction testing showed that the associations of age, education, marital status, wealth, parenthood and employment with heavy drinking differ significantly between males and females. Sex‐stratified estimates for moderate and low‐risk drinking follow a broadly consistent direction, though formal interaction tests were not conducted for those contrasts. There is therefore the need for the development of sex‐sensitive interventions tailored to address the specific drivers of drinking among Ghanaian males and females. These interventions should specifically prioritise heavy drinking in older males and monitor alcohol use in mothers, while leveraging the protective effects of marriage through couple‐ or family‐based approaches. Further research should also be conducted to examine the mediating roles of cultural norms, peer influence and accessibility of alcohol in alcohol use, so as to explain the sex differences observed in this study. Longitudinal studies are also needed to establish causal pathways.

## Author Contributions

**Christian Chukwuma Ndu** contributed to the conceptualisation of the study, analysis and interpretation of data and drafted and substantially revised paper. **Chioma Shallom Ndu** contributed to the conceptualisation of the study, interpretation of data and drafted and substantially revised paper. **Christa Osei‐Mensah** drafted and revised the paper. **Adusei Bofa** analysed, drafted and substantially revised paper. **Georgina Afoakwah** contributed to the conceptualisation of the study, and drafted and substantially revised paper.

## Funding

The authors have nothing to report.

## Ethics Statement

The authors have nothing to report.

## Consent

The authors have nothing to report.

## Conflicts of Interest

The authors declare no conflicts of interest.

## Data Availability

The data that support the findings of this study are openly available in Ghana Statistical Service Microdata Catalog at https://microdata.statsghana.gov.gh/index.php/home, reference number GHA_2022_DHS_v01_M.
